# Magnetic Resonance Imaging and Gait Analysis Indicate Similar Outcomes Between Yucatan and Landrace Porcine Ischemic Stroke Models

**DOI:** 10.3389/fneur.2020.594954

**Published:** 2021-01-21

**Authors:** Sydney E. Sneed, Kelly M. Scheulin, Erin E. Kaiser, Madison M. Fagan, Brian J. Jurgielewicz, Elizabeth S. Waters, Samantha E. Spellicy, Kylee J. Duberstein, Simon R. Platt, Emily W. Baker, Steven L. Stice, Holly A. Kinder, Franklin D. West

**Affiliations:** ^1^Regenerative Bioscience Center, University of Georgia, Athens, GA, United States; ^2^Department of Animal and Dairy Science, University of Georgia, Athens, GA, United States; ^3^Biomedical and Health Sciences Institute Neuroscience Program, University of Georgia, Athens, GA, United States; ^4^Department of Small Animal Medicine and Surgery, University of Georgia, Athens, GA, United States; ^5^Aruna Bio, Inc., Athens, GA, United States

**Keywords:** ischemic stroke, swine, porcine (pig) model, pig model, magnetic resonance imaging, gait analysis

## Abstract

The Stroke Therapy Academic Industry Roundtable (STAIR) has recommended that novel therapeutics be tested in a large animal model with similar anatomy and physiology to humans. The pig is an attractive model due to similarities in brain size, organization, and composition relative to humans. However, multiple pig breeds have been used to study ischemic stroke with potentially differing cerebral anatomy, architecture and, consequently, ischemic stroke pathologies. The objective of this study was to characterize brain anatomy and assess spatiotemporal gait parameters in Yucatan (YC) and Landrace (LR) pigs pre- and post-stroke using magnetic resonance imaging (MRI) and gait analysis, respectively. Ischemic stroke was induced via permanent middle cerebral artery occlusion (MCAO). MRI was performed pre-stroke and 1-day post-stroke. Structural and diffusion-tensor sequences were performed at both timepoints and analyzed for cerebral characteristics, lesion diffusivity, and white matter changes. Spatiotemporal and relative pressure gait measurements were collected pre- and 2-days post-stroke to characterize and compare acute functional deficits. The results from this study demonstrated that YC and LR pigs exhibit differences in gross brain anatomy and gait patterns pre-stroke with MRI and gait analysis showing statistical differences in the majority of parameters. However, stroke pathologies in YC and LR pigs were highly comparable post-stroke for most evaluated MRI parameters, including lesion volume and diffusivity, hemisphere swelling, ventricle compression, caudal transtentorial and foramen magnum herniation, showing no statistical difference between the breeds. In addition, post-stroke changes in velocity, cycle time, swing percent, cadence, and mean hoof pressure showed no statistical difference between the breeds. These results indicate significant differences between pig breeds in brain size, anatomy, and motor function pre-stroke, yet both demonstrate comparable brain pathophysiology and motor outcomes post-stroke. The conclusions of this study suggest pigs of these different breeds generally show a similar ischemic stroke response and findings can be compared across porcine stroke studies that use different breeds.

## Introduction

Stroke is one of the leading causes of disability and death with recent studies indicating there are 33 million stroke survivors and 5.9 million stroke-related deaths across nearly 300 countries every year (1–3). Ischemic stroke survivors often suffer from severe cognitive, behavioral, and motor function deficits that drastically impact their quality of life and frequently require long-term rehabilitation and medical care (3). In the United States alone, stroke had a $103.5 billion economic impact associated with direct (e.g., hospitalization, healthcare provider visits) and indirect (e.g., missed work days, premature mortality) costs (4). The devastating individual and societal effects of stroke have led to numerous late stage clinical trials, which have resulted in only a limited number of FDA approved treatments (tissue plasminogen activator and endovascular clot retrieval) that are effective for <25% of ischemic stroke patients (5–8). To improve therapeutic translation from pre-clinical testing to clinical use, the Stroke Therapy Academic Industry Roundtable (STAIR) has recommended that novel therapeutics be tested in multiple species including a large animal model that has similar anatomy and physiology to humans as they are likely to be more predictive of human outcomes (9). However, the use of large animal models is not yet widely utilized due to challenges associated with specialized maintenance requirements and large interdisciplinary research teams (10). Despite these challenges, the pig ischemic stroke model has a number of unique advantages in brain anatomy and physiology that make it an attractive model to study stroke pathophysiology and test novel therapeutics (11–15).

Rodent ischemic stroke models are the most commonly used systems to study stroke and assess potential therapies (16). However, the size, organization, and composition of the pig brain are more comparable to the human brain relative to the rodent brain, which potentially makes the pig a more translatable model for studying ischemic stroke and stroke therapeutics (17). The average pig brain at 180 grams (g) is more analogous to the average 1,300 g human brain than the 10,g rodent brain (18). Having gyrencephalic brains that are comprised mostly of white matter, pigs also exhibit greater similarity in brain structure to humans opposed to rodents that have lissencephalic brains containing <15% white matter (18–20). In addition, anatomical and functional studies have revealed important similarities in the occipital cortex, somatosensory cortices, motor cortex, and prefrontal cortex using cytoarchitecture, electrophysiology, and neuronal tracing approaches to compare pig and human brains (19). Due to these similarities, the pig stroke model may demonstrate more predictive injury, recovery, and therapeutic responses relative to humans than rodent models, and may bridge the gap between pre-clinical studies and human clinical trials.

Recent studies in the pig stroke model have evaluated novel therapeutics and their effects on cellular and tissue damage, and functional deficits. Our research team has shown neural stem cell extracellular vesicle treatment led to decreased lesion volumes and intracranial hemorrhage, and improved white matter integrity, thus resulting in enhanced recovery of exploratory behavior and gait deficits compared to non-treated controls (13). Others have shown that selective hypothermia and epidural cooling led to neuroprotection and decreased infarct volume after acute ischemic stroke in the porcine model (21, 22). However, it is important to note that these and other pre-clinical studies have been performed in a wide variety of pig breeds (e.g., Landrace, Yucatan, and Yorkshire) that vary in size, genetics, and anatomy, thus raising the question whether the findings in these stroke studies are comparable due to inherent breed differences.

This is a critically important question as it has been previously established that mouse and rat strains show differences in cerebrovascular anatomy and resting-state networks that contribute to divergent stroke responses. Branching patterns and vessel diameter of major cerebral arteries, including the middle cerebral artery (MCA), have been shown to differ between rodent strains (23–25). These differences in vasculature directly influenced reperfusion and lesion volumes post-stroke. Unique collateralization between strains also affect cerebral ischemia severity, lesion evolution, and edema formation (26–28). Resting-state functional MRI studies have revealed significant differences in brain glucose metabolism between mouse strains in the frontal, cingulate, sensorimotor, and hypothalamus/thalamus networks, which are involved in cognitive, motor, and sensory behaviors (29). Variabilities in these major networks could impact functional recovery post-stroke, which may explain differences observed between strains in forced-swim and rotarod tests as well as impairments in light-dark cycle activity patterns, despite equivalent lesion volumes post-stroke (30, 31). Due to these relevant variations between rodent strains having been established, and recent review proposing that there may be neuroendovascular differences in large animal models as well (10), it is important to investigate the possibility that similar differences could exist between pig breeds.

The objective of this study was to characterize and compare gross neuroanatomical and functional differences between Yucatan (YC) and Landrace (LR) breeds pre- and acutely post-stroke. In the current study, we evaluated lesion size and diffusivity, cerebral volumetric changes, white matter integrity, and other pathophysiological responses post-stroke in both breeds. Additionally, we evaluated spatiotemporal gait parameters to determine if breed differences influenced functional outcomes post-stroke.

## Materials and Methods

### Animals

All work performed in this study was approved by the University of Georgia (UGA) Institutional Animal Care and Use Committee (IACUC) and in accordance with the National Institutes of Health Guide for the Care and Use of Laboratory Animals guidelines. Pigs were housed in a Public Health Service (PHS) and Association for Assessment and Accreditation of Laboratory Animal Care (AAALAC) approved facility. Ten YC biomedical pigs (three ovariectomized females and seven castrated males; mean age 16 months; mean weight 81.9 kg) and 13 LR-cross agriculture pigs (castrated males; mean age 6 months; mean weight 75.5 kg) were assessed for this study. YC pigs were purchased from Exemplar Genetics (Sioux Center, IA) and LR pigs were obtained from the UGA swine unit (Athens, GA). Predefined exclusion criteria included instances that would prevent the animal from reaching the 24-h MRI or 2-day gait collection events (such as death or euthanasia due to severe self-inflicted injuries, inability to thermoregulate, uncontrolled seizure activity, and/or respiratory distress) and confounding infections at the incision. One LR pig (2W278) and four YC pigs (A1401 + A1645 + A1382 + A1589) were initially included and later excluded due to exclusion criteria (Supplementary Table 1).

The sample size for this study was determined by a power calculation based on our routine use of the MCAO model with lesion volume changes by MRI imaging being the primary endpoint. The power analysis was calculated using a two-tailed ANOVA test, α = 0.05, and an 80% power of detection effect size of 1.19 and a standard deviation of 44.63. Both males and females were included in this study. At an average of 6 months for Landrace-cross (LR) and 16 months for Yucatan (YC) pigs, both groups of animals are sexually mature and considered young adults. To keep the size comparable between breeds and to permit the use of MRI on the larger LR pig, weight was used as the matching factor. The size of the animal is important to consider for gait parameters, where a much heavier or larger animal may present different gait parameters such as velocity, stride length, and pressure distribution between hooves. Further, we are limited in the size of pig that we can use in our MRI scanner. The MRI table is only approved to hold up to 250 lbs, and LR pigs can reach over 500 lbs at 12 months of age.

### Permanent Middle Cerebral Artery Occlusion

To induce ischemic stroke, a permanent MCAO was performed as previously described (32). Briefly, a right frontotemporal craniectomy and orbital rim ostectomy with a partial zygomatic arch resection was performed. Bipolar cautery forceps were used to permanently occlude the right distal MCA and all associated branches. Musculature and epidermal layers of the surgical site were re-apposed and sutured following occlusion. After surgery, pigs were returned to their pens, extubated, and monitored every 4 h until vitals returned to baseline. The ipsilateral eye may be impacted up to 48 h post-surgery by minor swelling around the surgical site in some animals and may contribute to impaired vision. After acute swelling subsides, eyesight returns to normal. Heart rate, respiratory rate, and temperature were recorded at each time point. Banamine (2.2 mg/kg) was administered IM for postoperative pain, acute inflammation, and fever management every 12 h for the first 24 h, and every 24 h for 3 days post-stroke (13, 33).

### Magnetic Resonance Imaging

MRI was performed pre-stroke and 1-day post-stroke on a GE Signa HDx 3.0 Tesla scanner. MRI of the cranium was performed using an 8-channel torso coil with the pig positioned in supine recumbency, as previously described (13). Standard multiplanar MRI sequences were acquired including 3D Fast SPoiled GRadient echo (FSPGR) T1Weighted (T1W), Fast Spin Echo (FSE) T2Weighted (T2W), T2Weighted Fluid Attenuated Inversion Recovery (T2FLAIR), Spin Echo (SE) Diffusion Weighted Imaging (DWI), and SE Diffusion Tensor Imaging (DTI). Apparent diffusion coefficient (ADC) maps were generated from DWI sequences. Fractional anisotropy (FA) maps were generated from DTI sequences using Oxford's FMRIB diffusion toolbox (FDT) (FSL, University of Oxford, UK) (34–36). Perfusion weighted imaging (PWI) was not able to be performed as a power injector for the injection of contrast agent was not available.

### MRI Analysis

T2FLAIR, T2W, DWI, and ADC maps underwent region of interest (ROI) analysis using Osirix software (Version 10.0.5) (37). FMRIB's automated segmentation tool (FAST) was used to analyze T1W sequences for cerebral tissue composition (38, 39). FA maps were analyzed using ImageJ software (representative images of manual ROI analysis for YC and LR pigs can be seen in Supplementary Figure 1) (Version 2.0.0-rc-69/1.52p) (40). Post-stroke FA values in the internal capsules were expressed as a percent change in the ipsilateral hemisphere relative to the contralateral hemisphere. Cytotoxic edema consistent with ischemic stroke was confirmed at 1-day post-stroke by comparing corresponding hyperintense regions in DWI sequences and hypointense regions in ADC maps.

As described by Webb et al., a single researcher performed manual ROI analysis on T2W sequences to calculate hemisphere and cerebellum volumes, while ischemic lesion volumes were calculated via ADC maps as previously described by Gerriets et al. to control for the space-occupying effect of brain edema (13, 41). Corrected lesion volumes were calculated according to the following formula modified from Loubinoux et al. in which *LV*^*c*^ and *LV*^*u*^ indicate corrected and uncorrected lesion volume, respectively, and *HV*_*c*_ and *HV*_*i*_ indicate volume of the contralateral and ipsilateral hemisphere, respectively (42).

$$\begin{array}{l} LV^{c}=HV_{c}+HV_{i}-\left(HV_{c}+HV_{i}-LV^{u}\right)\times \frac{HV_{c}+HV_{i}}{2HV_{c}} \end{array}$$

ADC was utilized to measure the diffusivity of the ischemic lesion. Average ADC values were calculated for each coronal slice and changes in mean ADC value of the ipsilateral hemisphere were expressed as a percentage change relative to the contralateral hemisphere.

Lateral ventricle compression was measured utilizing a similar ROI method to compare the volume of the contralateral lateral ventricle to that of the ipsilateral lateral ventricle to compute a percent change in ventricular volume. Analysis of midline shift (MLS), caudal transtentorial herniation (CTH), and foramen magnum herniation (FMH) was performed on T2W sequences as previously described by Spellicy et al. (14, 43, 44). Briefly, MLS analysis was completed in the axial plane by first identifying the ideal midline and then, at the level of the septum pellucidum, measuring the deviation of the actual midline from the ideal midline. Herniation analysis was performed on midsagittal T2W sequences using previously identified landmarks: the dorsal aspect of the cribriform plate, dorsal aspect of the dorsal sella turcica, rostral aspect of the osseous tentorium cerebelli, caudoventral aspect of the occipital bone (dorsal margin of the foramen magnum), and caudal aspect of the basioccipital bone (ventral aspect of the foramen magnum) (14, 44). These landmarks were used to create three linear measurements: the skull length line (SLL), transtentorial line to the rostral most point along the ventral aspect of the cerebellum (TTX), and the most caudal point on the ventral aspect of the cerebellum to the foramen magnum line (FMX). Caudal transtentorial herniation (CTH) was quantified as TTX/SLL, such that CTH = TTX/SLL. Foramen magnum herniation (FMH) was quantified as FMX/SLL, such that FMH = FMX/SLL.

### Gait Analysis

Gait data was collected pre- and post-stroke using GAITFour software (Version 4.9x5; GaitRite Quadruped Gait Analysis System, NJ) as previously described (13). For this study, we will report the major parameters: velocity, cadence, cycle time, mean hoof pressure, percent of stride spent in swing phase, and stride length for all four limbs [left front (LF), right front (RF), left hind (LH), right hind (RH)]. Pre-stroke gait parameters were collected after a training period in which the animals were acclimated to the gait testing room and data collection procedure. This was done to ensure animals moved across the pressure sensitive gait mat at a consistent pace with no distractions. After the training period, gait parameters were recorded on three separate days to obtain an average pre-stroke measurement per animal, per parameter. Gait data was also collected 2-days post-stroke. To obtain an average for each parameter post-stroke, all animals were required to travel across the gait mat at a consistent two-beat pace until five “good” runs (runs with a minimum of 12 consecutive footfalls or three gait cycles and no external distractions) for each trial were collected. A video showing what researchers observed using the GAITfour software during gait collection is shown in Supplementary Movie 1 and Supplementary Video depicting a categorically “good” run can be seen in Supplementary Movie 2. Only data from five runs were used to calculate post-stroke performance. Two trained observers with a minimum of 3 years of experience oversaw all gait data collection. Post-stroke gait data was then normalized to each individual pig's pre-stroke data for each given parameter to acquire a percent change for each parameter after stroke. All gait raw data was automatically analyzed by the GAITfour software and thus removing human error and subjectivity.

### Statistical Analysis

Data was analyzed with JMP Pro (Version 14.1.0; SAS Institute Inc., Cary, NC) and GraphPad Prism (Version 8.4.0; San Diego, CA). Normal Gaussian distribution was confirmed for each data set using the Shapiro-Wilk's test with *P*-values ≥ 0.05 considered normally distributed. Statistical difference between pig breeds was determined by using a Welch-Satterthwaite's *t*-test of two independent samples. Comparisons where *P*-values were ≤ 0.05 were considered significantly different. Gait data was not corrected for multiple comparisons. Data is reported as mean ± standard deviation.

## Results

### Pre-stroke MRI Reveals Innate Neuroanatomical Variations Between Breeds

**Figure 1:**
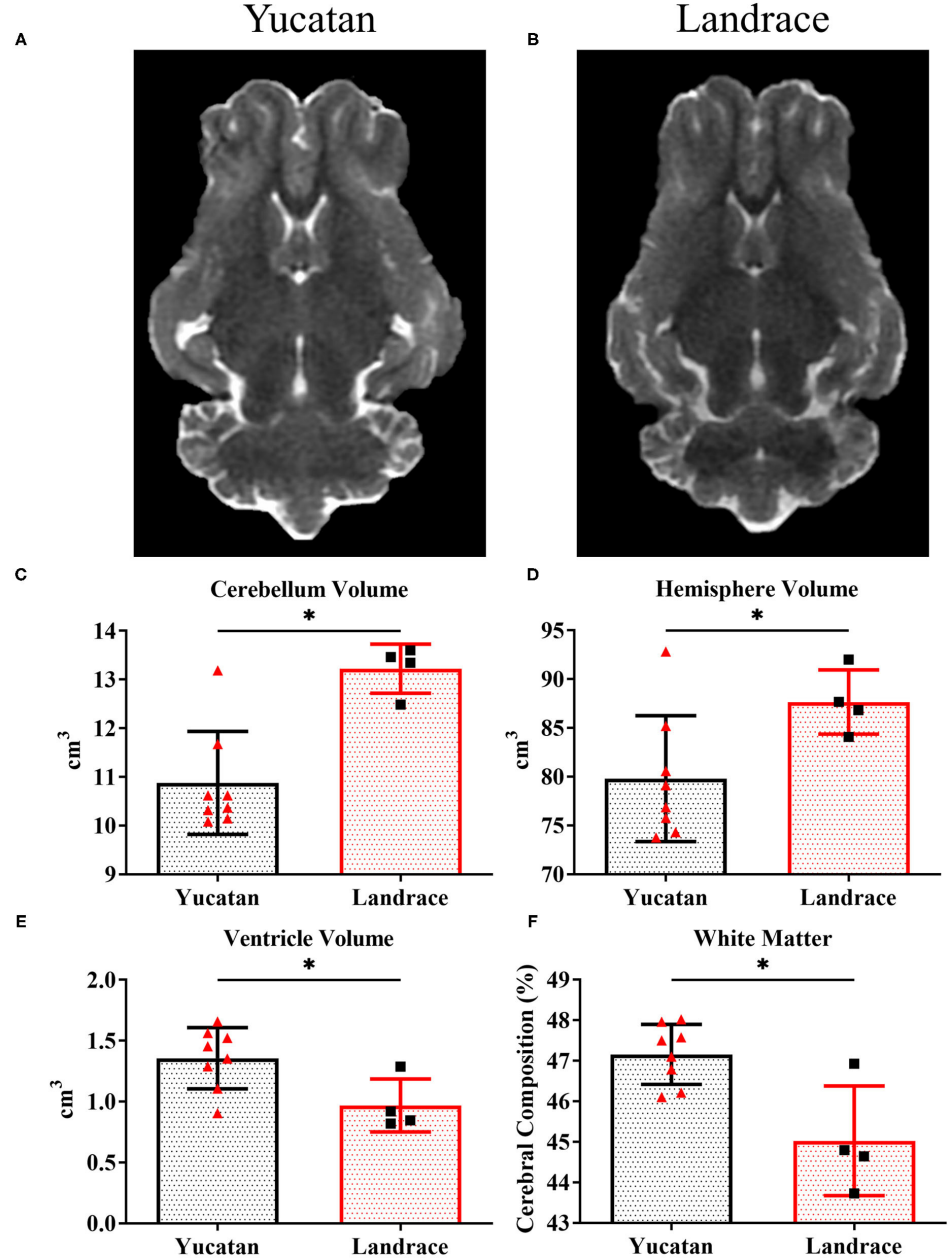
YC and LR pigs have inherently different cerebral anatomy and composition. Pre-stroke T2W sequences for YC **(A)** and LR **(B)** pigs showed significant (*p* < 0.05) differences in cerebellar volume **(C)**, hemisphere volume **(D)**, lateral ventricle volume **(E)**, and white matter composition **(F)**. ^*^Significant difference between breeds.

**Figure 2:**
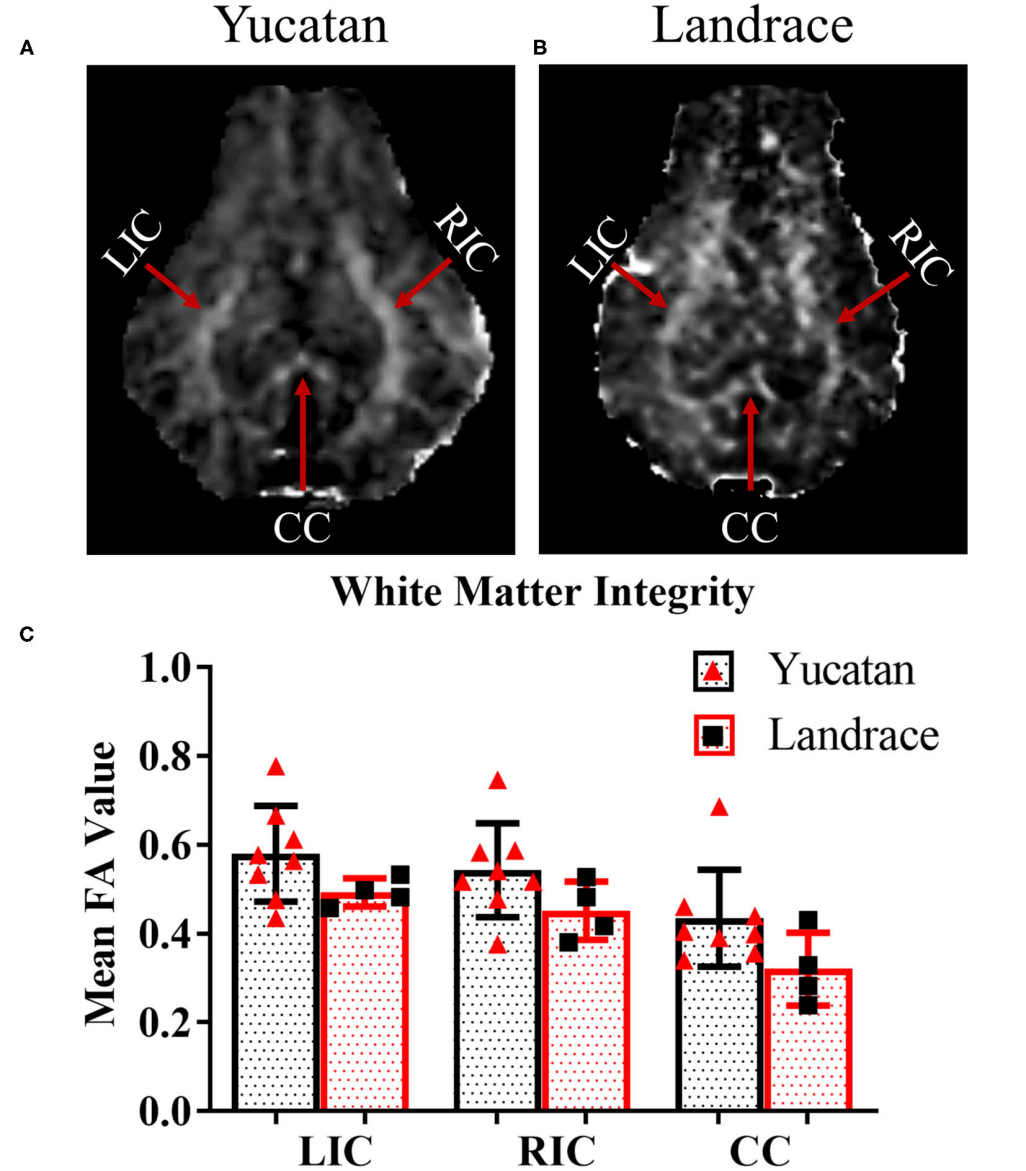
White matter integrity in major tracts of YC and LR pigs are comparable. Pre-stroke FA maps for YC **(A)** and LR **(B)** pigs showed no significant differences in mean FA values in major white matter tracts **(C)** LIC, left internal capsule; RIC, right internal capsule; and CC, corpus callosum. Red arrows indicate major tracts as noted in **(A,B)**. *Significant difference between breeds.

To determine if there were comprehensive differences in brain size, ventricular space, and white matter between YC (Figure 1A) and LR (Figure 1B) breeds, pre-stroke MRI structural sequences were analyzed. LR pigs exhibited a significantly (*p* < 0.05) larger cerebellum volume (13.2 ± 0.5 cm^3^ vs. 10.9 ± 1.1 cm^3^; Figure 1C) and total hemisphere volume (87.6 ± 3.3 cm^3^ vs. 79.8 ± 6.5 cm^3^; Figure 1D, respectively) than YC pigs. However, YC pigs possessed a larger lateral ventricle volume (*p* < 0.05) than LR pigs (1.4 ± 0.3 cm^3^ vs. 0.97 ± 0.2 cm^3^; Figure 1E). Additionally, YC pigs had a greater percentage of cerebral white matter (*p* < 0.05) than LRs (47.1 ± 0.7% vs. 45.0 ± 1.4%; Figure 1F). However, no significant differences were observed in mean FA values between YC (Figure 2A) and LR (Figure 2B) pigs pre-stroke for major white matter tracts (indicated by corresponding red arrows; Figures 2A,B) including the left internal capsule (LIC), right internal capsule (RIC), and corpus collosum (CC; Figure 2C). These differences between YC and LR pigs suggest the animals exhibit innate gross neuroanatomical differences.

### Pre-stroke Gait Biomechanics Are Variable Between Breeds

**Figure 3:**
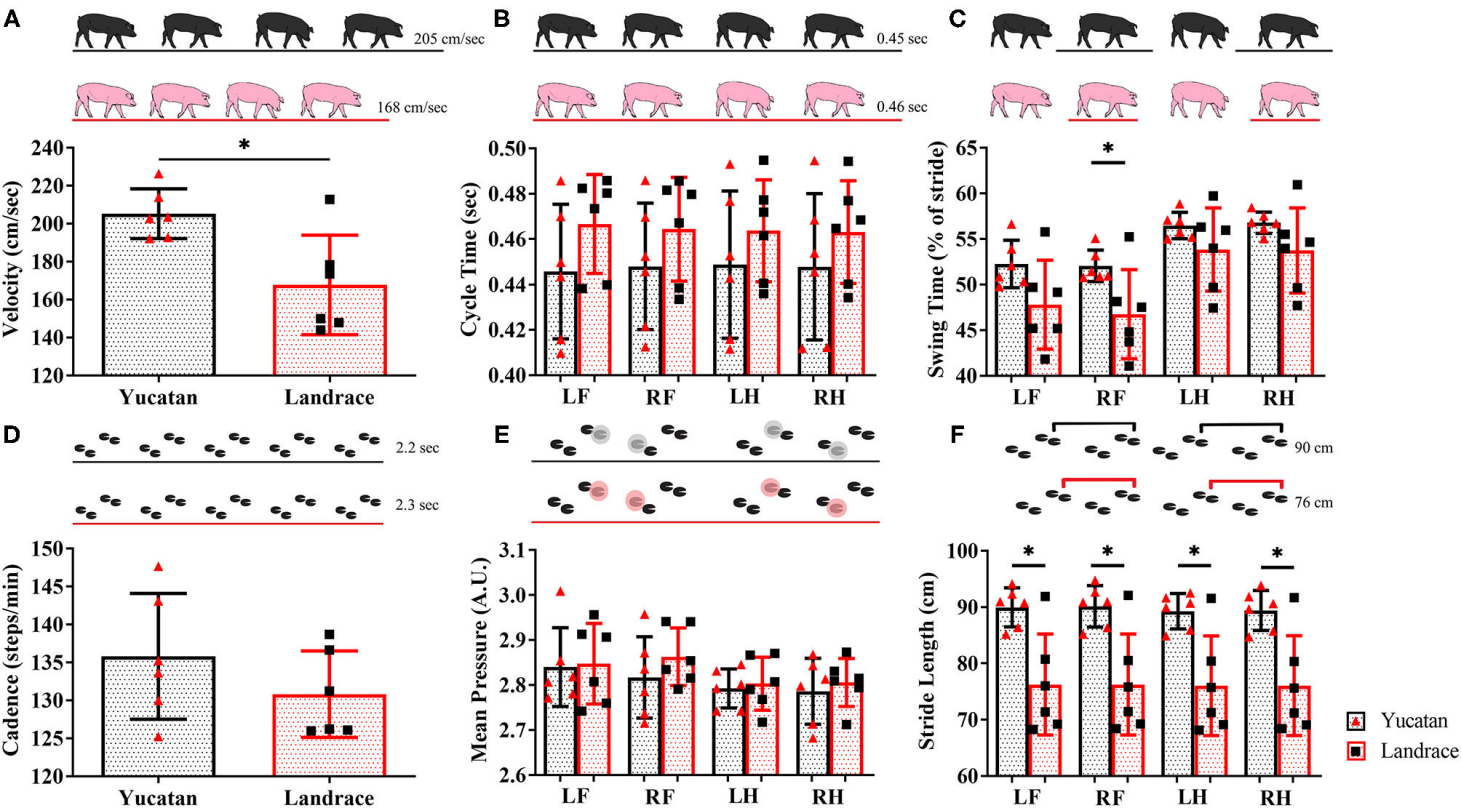
Innate differences in gait exist between YC and LR pigs. Pre-stroke there were no significant (*p* > 0.05) differences between YC (black) and LR (pink) pigs in cycle time **(B)**, cadence **(D)**, and mean hoof pressure for all four limbs **(E)**. However, there were significant (*p* < 0.05) differences between breeds in velocity **(A)**, swing time **(C)**, and stride length in all four limbs **(F)**. See Table 1 for corresponding data for each graph. LF, left front; RF, right front; LH, left hind, RH: right hind. *Significant difference between breeds.

Spatiotemporal gait parameters were assessed in pre-stroke YC and LR animals to determine if innate ambulation differences existed between these two breeds. There were no significant differences between YC (black pigs) and LR (pink pigs) pigs in cycle time (Table 1; Figure 3B), cadence (Table 1; Figure 3D), and mean hoof pressure (Table 1; Figure 3E). However, YC pigs showed a significantly (*p* < 0.05) higher velocity (Table 1; Figure 3A) and longer stride length for all four limbs (Table 1; Figure 3F) as compared to LR pigs. Interestingly, YC pigs showed a significantly (*p* < 0.05) higher percentage of stride in the swing phase only for the RF limb, but there was no significant difference between YC and LR pigs for all other limbs (Table 1; Figure 3C). These results suggest that there are differences in gait between the breeds prior to neural injury.

**Table 1:** Pre-stroke gait parameters in Yucatan and Landrace pigs.

*Denotes difference between breeds	**Yucatan**	**Landrace**
**Velocity (cm/s)**	205.2 ± 13.1*	167.7 ± 26.3*
**Cycle Time (s)**		
Left Front	0.45 ± 0.03	0.47 ± 0.02
Right Front	0.45 ± 0.03	0.46 ± 0.02
Left Hind	0.45 ± 0.03	0.46 ± 0.02
Right Hind	0.45 ± 0.03	0.46 ± 0.02
**Swing Time (% of stride)**		
Left Front	52.3 ± 2.6	47.8 ± 4.9
Right Front	52.1 ± 1.7*	46.7 ± 4.9*
Left Hind	56.4 ± 1.4	53.8 ± 4.6
Right Hind	56.8 ± 1.2	53.7 ± 4.7
**Cadence (steps/min)**	135.8 ± 8.3	130.8 ± 5.7
**Mean Pressure (A.U.)**		
Left Front	2.8 ± 0.09	2.8 ± 0.09
Right Front	2.8 ± 0.09	2.9 ± 0.06
Left Hind	2.8 ± 0.04	2.8 ± 0.06
Right Hind	2.8 ± 0.07	2.8 ± 0.05
**Stride Length (cm)**		
Left Front	89.9 ± 3.5*	76.3 ± 9.0*
Right Front	90.1 ± 3.7*	76.3 ± 9.0*
Left Hind	89.3 ± 3.1*	76.0 ± 8.9*
Right Hind	89.4 ± 3.5*	76.1 ± 8.9*

### Neuroanatomical and Physiological Responses to Stroke Are Comparable Between Breeds

**Figure 4:**
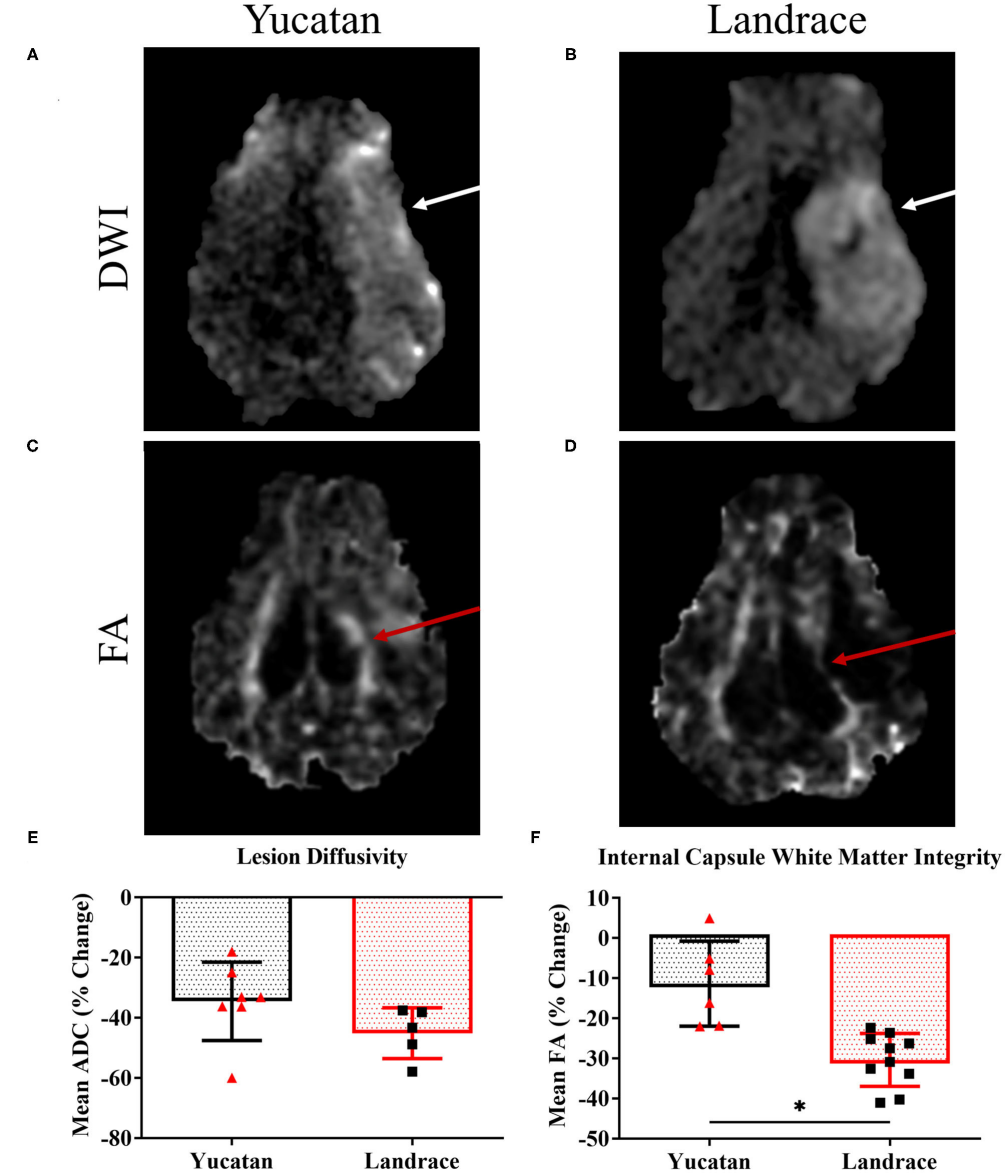
YC and LR pigs show similarities in lesion diffusivity 1-day post-stroke. DWI sequences in YC **(A)** and LR **(B)** pigs showed hyperintense lesions (white arrows) 1-day post-stroke. FA maps showed areas of white matter disruption (red arrows) in the internal capsule of YC **(C)** and LR pigs **(D)**. Although there was no significant (*p* < 0.05) difference in lesion diffusivity **(E)**, there was a significant (*p* < 0.05) difference in loss of internal capsule FA post-stroke **(F)**. *Significant difference between breeds.

DWI sequences for both YC and LR pigs, revealed a hyperintense lesion (Figures 4A,B, respectively) within the right hemisphere 1-day post-stroke. YC and LR pigs showed similar decreases in lesion diffusivity with no significant difference observed between YC and LR pigs (−34.5 ± 13.0% vs. −45.2 ± 8.4%, respectively; Figure 4E). However, significant differences in white matter integrity between YC (Figure 4C) and LR (Figure 4D) pigs were observed with LR pigs exhibiting a significantly (*p* < 0.05) larger decrease in white matter integrity as compared to YC pigs (−30.4 ± 6.6% vs. −11.4 ± 10.6%; Figure 4F).

**Figure 5:**
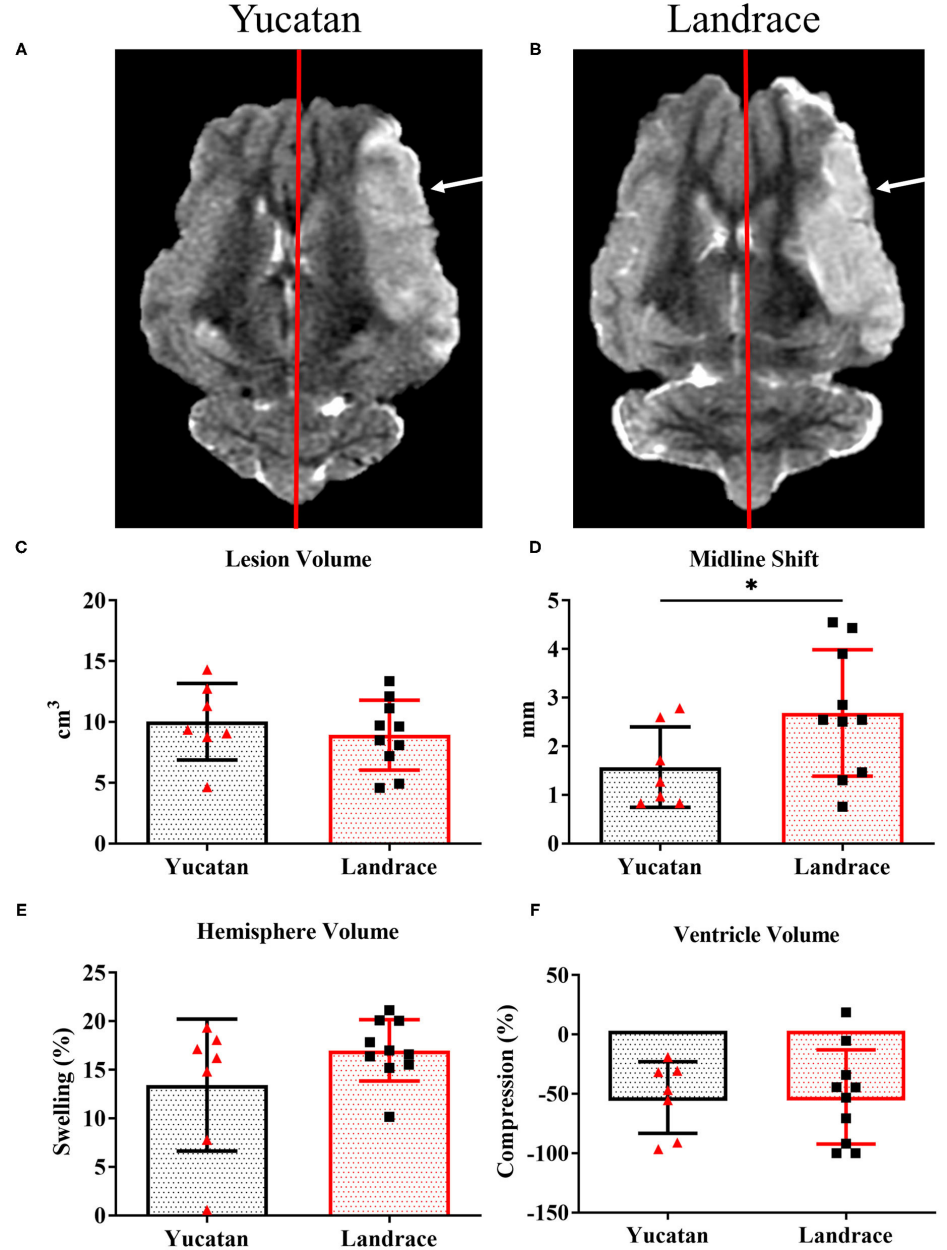
Most pathophysiological changes of the brain 1-day post-stroke were comparable between YC and LR pigs. T2W YC **(A)** and LR **(B)** sequences exhibit similar pathologies 1-day post-stroke with no differences in lesion volume (**C**; white arrows), hemispheric swelling **(D)**, and ventricle compression **(E)**. However, LR pigs show a significantly larger MLS (natural midline represented by red line) as compared to YC pigs **(F)**. *Significant difference between breeds.

**Figure 6:**
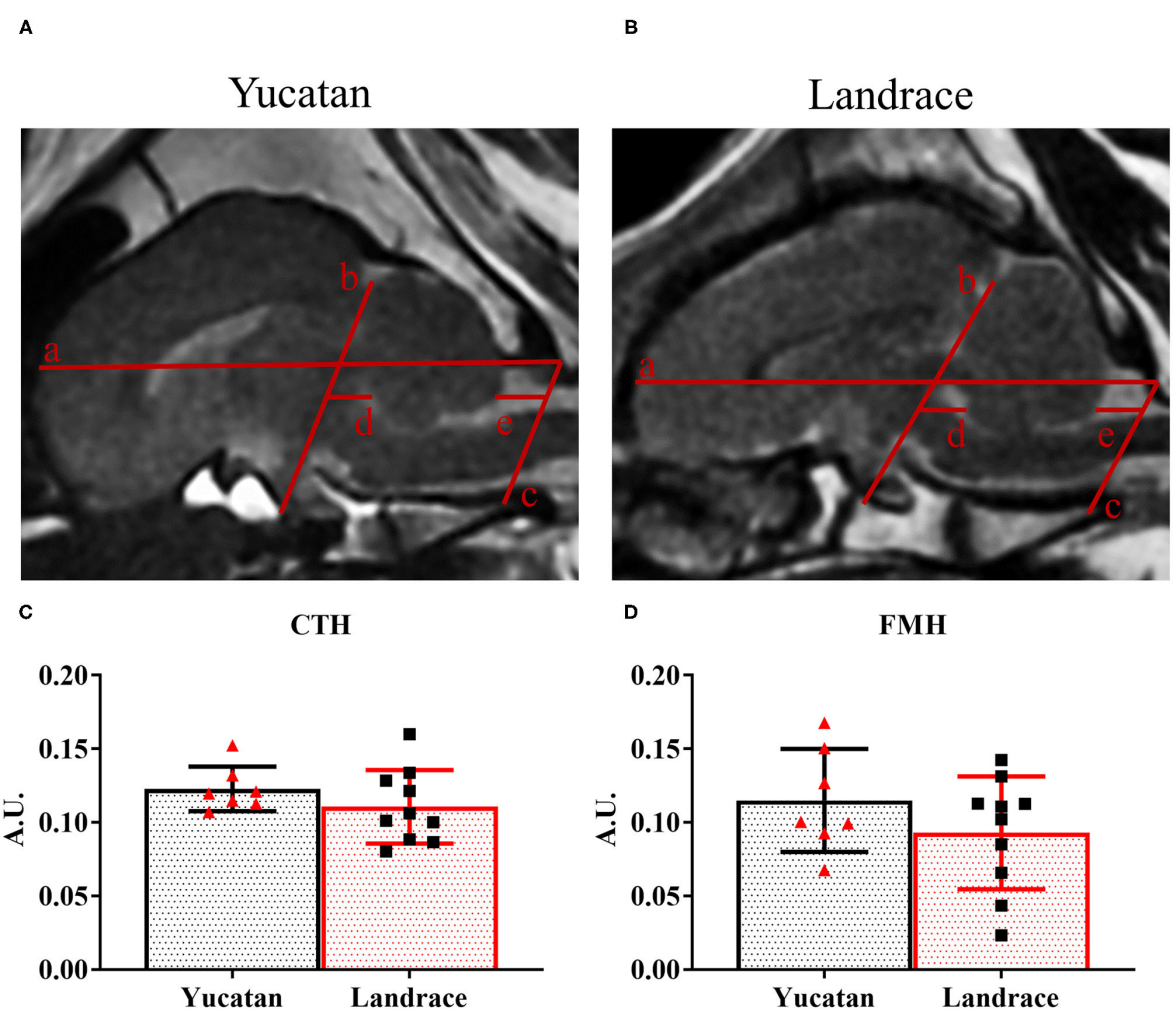
YC and LR pigs have equivalent levels of cerebellar herniation post-stroke. Mid-sagittal T2W images showed YC **(A)** and LR **(B)** pigs have similar levels of cerebellar herniation (red lines) 1-day after induction of ischemic stroke. Neither the degree of caudal transtentorial herniation (CTH = TTX/SLL; **C**) nor that of foramen magnum herniation (FMH = FMX/SLL; **D**) is significantly different between pigs. a: SLL, b: TTL, c: FML, d: TTX, e: FMX. *Significant difference between breeds.

To determine possible differences in ischemic lesioning and swelling between YC and LR pigs, MRI analysis was performed post-stroke to compare lesion volume, hemispheric swelling, ventricle compression, MLS, and herniation. Analysis of structural MRI sequences 1-day post-stroke revealed no significant difference between YC (Figure 5A) and LR (Figure 5B) pigs in lesion volume (10.0 ± 3.1 cm^3^ vs. 8.9 ± 2.9 cm^3^; Figure 5C), degree of hemispheric swelling (13.4 ± 6.8% vs. 17.0 ± 3.2%; Figure 5D), or degree of ventricle compression (−53.1 ± 30.2% vs. −52.6 ± 39.7%; Figure 5E). Additionally, there was no significant difference between breeds when lesion volume was normalized to hemispheric volume (YC 59.0 ± 19.6% vs. LR 56.5 ± 14.5%). However, LR pigs did show a significantly (*p* < 0.05) larger MLS as compared to YC pigs (2.7 ± 1.3 mm vs. 1.6 ± 0.8 mm; Figure 5F). Cerebellar herniation analysis of YC (Figure 6A) and LR (Figure 6B) pigs using both CTH (0.12 ± 0.02 vs. 0.11 ± 0.03; Figure 6C) and FMH (0.11 ± 0.04 vs. 0.09 ± 0.04; Figure 6D) approaches showed no significant differences. While there were significant differences found in white matter integrity and midline shift parameters post-stroke, collectively the data revealed YC and LR pigs respond to stroke in a comparable manner.

### Post-stroke Functional Deficits Are Similar Between Breeds

**Figure 7:**
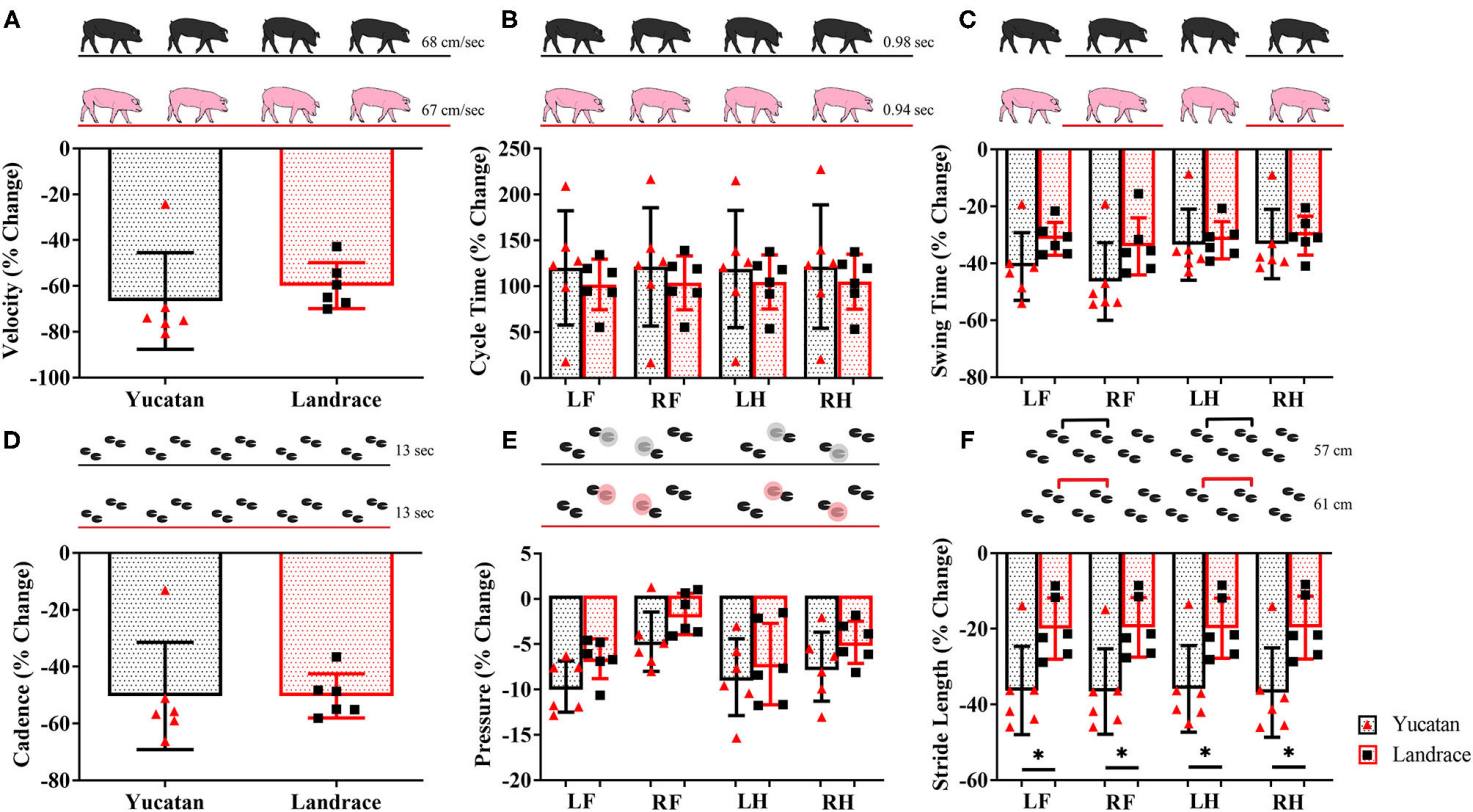
The majority of motor function changes post-stroke are comparable between YC and LR pigs. There were no significant differences between breeds in velocity **(A)**, cycle time **(B)**, swing time **(C)**, cadence **(D)**, and mean hoof pressure for all four limbs **(E)**. However, there was a significant (*p* < 0.05) difference in stride length between YC and LR pigs **(F)**. See Table 2 for corresponding data for each graph. LF, left front; RF, right front; LH, left hind; RH, right hind. *Significant difference between breeds.

Gait data was collected 2-days post-stroke for LR and YC pigs. Between YC and LR pigs there were no significant differences in change of velocity (Table 2; Figure 7A), cycle time (Table 2; Figure 7B), swing percent (Table 2; Figure 7C), cadence (Table 2; Figure 7D), and mean hoof pressure (Table 2; Figure 7E). However, YC pigs exhibited a significantly (*p* < 0.05) larger decrease in stride length as compared to LR pigs (Table 2; Figure 7F). Percent change in stride length was significantly different between YC and LR breeds, however overall, this data suggests that both YC and LR pigs respond comparably to stroke in terms of functional gait parameters.

**Table 2:** Post-stroke gait parameters in Yucatan and Landrace pigs.

*Denotes difference between breeds	**Yucatan**	**Landrace**
**Velocity (% change)**	−66.6 ± 21.1	−59.8 ± 10.1
**Cycle Time (% change)**		
Left Front	119.8 ± 62.3	101.8 ± 27.7
Right Front	121.1 ± 64.5	103.6 ± 29.4
Left Hind	118.7 ± 63.9	104.5 ± 29.5
Right Hind	121.4 ± 67.2	104.9 ± 29.9
**Swing Time (% change)**		
Left Front	−41.1 ± 4.9	−31.4 ± 2.4
Right Front	−46.4 ± 5.6	−34.0 ± 4.1
Left Hind	−33.5 ± 5.1	−31.9 ± 2.7
Right Hind	−33.2 ± 5.0	−30.3 ± 2.8
**Cadence (% change)**	−50.3 ± 18.9	−50.3 ± 7.8
**Mean Pressure (% change)**		
Left Front	−9.7 ± 2.8	−6.6 ± 2.2
Right Front	−4.7 ± 3.3	−1.7 ± 2.3
Left Hind	−8.6 ± 4.2	−7.2 ± 4.5
Right Hind	−7.5 ± 3.8	−4.8 ± 2.3
**Stride Length (% change)**		
Left Front	−36.3 ± 11.7*	−19.9 ± 8.1*
Right Front	−36.6 ± 11.3*	−19.6 ± 7.9*
Left Hind	−35.9 ± 11.4*	−19.8 ± 8.0*
Right Hind	−36.9 ± 11.8*	−19.7 ± 8.3*

## Discussion

In the current study, we investigated breed-based differences between YC biomedical pigs and LR commercial pigs before and after ischemic stroke using neuroimaging and motor function analyses. Pre-ischemic stroke MRI analysis of gross anatomical brain volumetrics and cerebral composition analysis and spatiotemporal gait parameters showed significant differences between breeds. This led us to further question if these detected differences could affect the stroke response between pig breeds. However, YC and LR pigs showed comparable lesion volumes, intracerebral swelling, and cerebellar herniation after stroke. In addition, both breeds showed similar impairments in velocity, cadence, cycle time, swing time, and mean hoof pressure. These results indicated that although there are significant differences between pig breeds in brain size and composition and motor function pre-stroke, both demonstrate comparable cerebral damage and motor deficits in response to ischemic stroke. Conclusions from this study suggest that findings can be compared across porcine stroke studies of these two commonly used swine breeds.

MRI analysis in the current study demonstrated that stroke in YC and LR pigs were highly comparable with 75% (six out of eight) of evaluated parameters showing no statistical difference. Lesion characteristics, such as volume and diffusivity, were not different between breeds suggesting similar pathophysiological (e.g., ischemia, excitotoxicity, cytotoxic edema) responses. Furthermore, comparable levels of hemisphere swelling, ventricle compression, and cerebellar herniation indicated similar stroke progression between breeds. Edema formation is a serious clinical complication of ischemic stroke and can lead to mechanical compression of adjacent brain structures, herniation, and death (15, 45). Therefore, consistency between models in parameters such as hemispheric swelling and ventricle compression are important in order to accurately evaluate stroke severity and novel therapeutic efficacy. Differences were seen in internal capsule FA and MLS post-stroke when comparing breeds. FA, which measures the directionality of molecular displacement by diffusion, is used to infer white matter tract integrity. FA is often decreased in major white matter tracts post-stroke due to microstructural damage and loss of structural integrity resulting from local tissue damage and/or retrograde axonal degeneration (46, 47). The asymmetrical decrease in FA may potentially be due to differences in white matter resilience, such that YC white matter is more refractory to injury. However, further studies are needed to explore this working hypothesis. Additionally, the MLS was greater in LR than YC pigs, despite both breeds exhibiting similar lesion volumes and hemispheric swelling percentages. These differences suggest that the variation in MLS response between breeds may be due to structural differences in the calvarium. The skull size and snout length of the YC pig (and other biomedical breeds such as Sinclair and Göttingen) is considerably smaller and shorter than domestic farm breeds (e.g., Landrace and Yorkshire) (48). This has resulted in YC pigs also having narrower nasal cavities and smaller sinuses (49). Furthermore, it has been established that different breeds of swine have whole crania differences as well as subset features, including the parietal, the basicranium, the angle of the nasal and the zygomatic, using 3D geometric morphometrics analysis (50). Consequently, the larger and more elongated skull of the LR pigs may allow for a greater shift in the midline as compared to a more rounded, compact cranium as seen in YC pigs (51). This proposed hypothesis could explain the differences in MLS despite equivalent swelling, herniation, lesion size, and functional responses seen between the breeds.

Gait analysis in the current study shows that the stroke motor function response in YC and LR pigs is highly comparable, with 83% (five out of six) of evaluated gait changes showing no significant difference between breeds. Changes in motor function are used to assess the severity of stroke and potential for recovery in the clinical setting. Damage to the motor cortices and associated descending tracts due to the ischemic attack results in muscle weakness and often hemiparesis of the infarct side (52). While there are kinematic differences between the bipedal gait of humans and the quadrupedal gait of pigs, several of the parameters which we assessed are also used in clinical practice; thus, can be used as a relevant measure of stroke progression and recovery between these two breeds. Ischemic stroke deficits in velocity, cycle time, cadence, swing phase, and hoof pressure were equivalent between breeds. Stride length was the only gait parameter found to be different between breeds. When comparing stride length changes due to stroke across studies, it is noteworthy that the changes in stride length for YC and LR animals in this study are comparable to previous studies (13). The similarities in stroke responses as measured by spatiotemporal gait parameters between these two breeds indicated stroke may impact key motor centers (e.g., primary motor, premotor, and primary somatosensory cortices) in a comparable manner.

Although stroke responses were comparable between breeds, YC and LR pigs exhibit differential gross brain anatomy and spatiotemporal gait patterns pre-stroke. These differences may be explained by unique anatomical variances observed between these biomedical and agriculture breeds. One potential reason that the stroke response was generally similar between breeds may in part be due to normalization of post-stroke data to pre-stroke data, which is done to account for individual variability in brain anatomy and gait. Inter-individual differences between porcine subjects can stem from unique behavioral or physical (e.g., height, weight) differences between animals. However, by individually normalizing post-stoke metrics (e.g., swing time, velocity) to pre-stroke metrics, we can account for individual differences and better assess stroke specific effects when comparing groups. Before normalization, there is greater variance seen for both breeds, with YC pigs generally having a higher variance than LR pigs. For example, non-normalized velocity 2-days post-stroke shows a SD of 42.4 m/s for YC pigs and 15.5 m/s for LR pigs. However, after individual normalization of the same data, the SD decreases by nearly 50% with SD showing a 21.1% velocity decrease for YC pigs and 10.1% velocity decrease for LR pigs. A similar pattern of decreased variance after normalization is observed for other gait parameters, such as cadence, step length, and swing time. Thus, normalization serves to decrease variance within a group by accounting for inter-individual differences and, consequently, increases the comparability of results between breeds and studies.

The STAIR recommendations suggested the use of large animal models over a decade ago, however large animal model use has remained limited with only a few stroke studies in dogs, sheep, and pigs (33, 53). Some of the most significant limiting factors in using the pig stroke model are cost and accessibility as compared to widely popular rodent models. The YC pig, while originally a meat producing animal from the Yucatan Peninsula, has now been employed in the United States mainly as a biomedical pig, which means these herds are generally closed, pure-bred, and intensively managed. In addition, YC pigs are typically poor breeders that produce small litters and are raised by only a select few purpose breed facilities, all of which make YCs more expensive. Contrasting to YC, commercially available agriculture breeds such as the LR are raised by hundreds of facilities nationwide and are extensively cross-bred to produce large litter sizes under less intensively managed systems to fulfill the meat-market demand at a relatively lower price point. This often makes the LR option more cost effective and accessible. Despite these advantages, LR pigs present formidable management issues as they often mature to well over 200 kgs (while YC are often <50 kg at sexual maturity), making it challenging to house these animals or utilize them in devices that have specific size and/or weight limitations such as MRI magnets. Therefore, the YC pig is often the preferred option when size limitations are an issue. The ability to use either breed could reduce the barrier of entry for groups looking to utilize the translational porcine ischemic stroke model.

For the first-time, this study demonstrated comparable stroke pathologies and motor function changes in two unique pig breeds: the YC biomedical pig and the LR agriculture pig. These results suggested that both models would yield comparable results in studies evaluating stroke pathophysiology or novel treatments. These findings also indicated that it is possible to compare results across pig ischemic stroke studies performed by different research teams. Despite these critical findings, it is important to note this study only compared MCAO induced ischemic stroke in two breeds. Additional studies using other common biomedical and agricultural pig breeds to evaluate outcomes and potential treatments are warranted. Nevertheless, this is an important first look into determining pig breed differences in stroke responses, which will become even more important as the stroke field moves forward with more studies in the translational pig model.

## Data Availability Statement

The raw data supporting the conclusions of this article will be made available by the authors, without undue reservation.

## Ethics Statement

The animal study was reviewed and approved by University of Georgia (UGA) Institutional Animal Care and Use Committee (IACUC). Written informed consent was obtained from the individual for the publication of any potentially identifiable images included in this article.

## Author Contributions

SSn and FW wrote and edited the manuscript. All co-authors reviewed the manuscript. SSn, KS, EK, MF, and BJ performed data analysis. KS, EK, MF, BJ, and EW performed pig work. SP performed MCAO. SSn, KS, EK, MF, BJ, SSp, KD, EWB, SLS, HK, and FW participated in study design. All authors contributed to the article and approved the submitted version.

## Conflict of Interest

EWB and SLS were employed by the company Aruna Bio. The remaining authors declare that the research was conducted in the absence of any commercial or financial relationships that could be construed as a potential conflict of interest.

## References

[1] Krishnamurthi RV Feigin VL Forouzanfar MH Mensah GA Connor M Bennett DA Global and regional burden of first-ever ischaemic and haemorrhagic stroke during 1990–2010: findings from the Global Burden of Disease Study 2010. Lancet Global Health. (2013) 1:e259–81. 10.1016/S2214-109X(13)70089-5 25104492 PMC4181351

[2] Feigin VL Forouzanfar MH Krishnamurthi R Mensah GA Connor M Bennett DA. Global and regional burden of stroke during 1990-2010: findings from the Global Burden of Disease Study 2010. Lancet. (2014) 383:245–54. 10.1016/S0140-6736(13)61953-4 24449944 PMC4181600

[3] Mozaffarian D Benjamin EJ Go AS Arnett DK Blaha MJ Cushman M. Heart disease and stroke statistics, 2015 update. Circulation. (2015) 131:e29–322. 10.1161/CIR.0000000000000152 25520374

[4] Girotra T Lekoubou A Bishu K Ovbiagele B. A contemporary and comprehensive analsyis of the costs of stroke in the United States. J Neurol Sci. (2019) 410:116643. 10.1016/j.jns.2019.116643 31927342

[5] Kernan WN Ovbiagele B Black HR Bravata DM Chimowitz MI Ezekowitz MD. Guidelines for the prevention of stroke in patients with stroke and transient ischemic attack: a guideline for healthcare professionals from the American Heart Association/American Stroke Association. Stroke. (2014) 45:2160–236. 10.1161/STR.0000000000000024 24788967

[6] Peña ID Borlongan C Shen G Davis W. Strategies to extend thrombolytic time window for ischemic stroke treatment: an unmet clinical need. J Stroke. (2017) 19:50–60. 10.5853/jos.2016.01515 28178410 PMC5307939

[7] Chen X Cheng X Zhang S Wu D. ADAMTS13: an emerging target in stroke therapy. Front Neurol. (2019) 10:772. 10.3389/fneur.2019.00772 31379722 PMC6650536

[8] Ringleb P Bendszus M Bluhmki E Donnan G Eschenfelder C Fatar M. Extending the time window for intravenous thrombolysis in acute ischemic stroke using magnetic resonance imaging-based patient selection. Int J Stroke. (2019) 14:483–90. 10.1177/1747493019840938 30947642

[9] Fisher M Feuerstein G Howells DW Hurn PD Kent TA Savitz SI. Update of the stroke therapy academic industry roundtable preclinical recommendations. Stroke. (2009) 40:2244–50. 10.1161/STROKEAHA.108.541128 19246690 PMC2888275

[10] Herrmann AM Meckel S Gounis MJ Kringe L Motschall E Mülling C. Large animals in neurointerventional research: a systematic review on models, techniques and their application in endovascular procedures for stroke, aneurysms and vascular malformations. J Cerebr Blood Flow Metab. (2019) 39:375–94. 10.1177/0271678X19827446 30732549 PMC6421248

[11] Baker EW Platt SR Lau VW Grace HE Holmes SP Wang L. Induced pluripotent stem cell-derived neural stem cell therapy enhances recovery in an ischemic stroke pig model. Sci Rep. (2017) 7:10075. 10.1038/s41598-017-10406-x 28855627 PMC5577218

[12] Lau VW Platt SR Grace HE Baker EW West FD. Human iNPC therapy leads to improvement in functional neurologic outcomes in a pig ischemic stroke model. Brain Behav. (2018) 8:e00972. 10.1002/brb3.972 29761021 PMC5943801

[13] Webb RL Kaiser EE Jurgielewicz BJ Spellicy S Scoville SL Thompson TA. Human neural stem cell extracellular vesicles improve recovery in a porcine model of ischemic stroke. Stroke. (2018) 49:1248–56. 10.1161/STROKEAHA.117.020353 29650593 PMC5916046

[14] Spellicy SE Kaiser EE Bowler MM Jurgielewicz BJ Webb RL West FD. Neural stem cell extracellular vesicles disrupt midline shift predictive outcomes in porcine ischemic stroke model. Transl Stroke Res. (2019) 11:776–88. 10.1007/s12975-019-00753-4 31811639 PMC7340639

[15] Kaiser EE Waters ES Fagan MM Scheulin KM Platt SR Jeon JH. Characterization of tissue and functional deficits in a clinically translational pig model of acute ischemic stroke. Brain Res. (2020) 1736:146778. 10.1016/j.brainres.2020.146778 32194080 PMC10671789

[16] Fluri F Schuhmann MK Kleinschnitz C. Animal models of ischemic stroke and their application in clinical research. Drug Design Dev Ther. (2015) 9:3445–54. 10.2147/DDDT.S56071 26170628 PMC4494187

[17] Kinder HA Baker EW West FD. The pig as a preclinical traumatic brain injury model: current models, functional outcome measures, and translational detection strategies. Neural Regen Res. (2019) 14:413–24. 10.4103/1673-5374.245334 30539807 PMC6334610

[18] Sauleau P Lapouble E Val-Laillet D Malbert CH. The pig model in brain imaging and neurosurgery. Animal. (2009) 3:1138–51. 10.1017/S1751731109004649 22444844

[19] Lind NM Moustgaard A Jelsing J Vajta G Cumming P Hansen AK. The use of pigs in neuroscience: modeling brain disorders. Neurosci Biobehav Rev. (2007) 31:728–51. 10.1016/j.neubiorev.2007.02.003 17445892

[20] Clouard C Meunier-Salaün M-C Val-Laillet D. Food preferences and aversions in human health and nutrition: how can pigs help the biomedical research? Animal. (2012) 6:118–36. 10.1017/S1751731111001315 22436160

[21] Zhang L Cheng H Shi J Chen J. Focal epidural cooling reduces the infarction volume of permanent middle cerebral artery occlusion in swine. Surg Neurol. (2007) 67:117–21; discussion 121. 10.1016/j.surneu.2006.05.064 17254860

[22] Mattingly TK Denning LM Siroen KL Lehrbass B Lopez-Ojeda P Stitt L. Catheter based selective hypothermia reduces stroke volume during focal cerebral ischemia in swine. J Neurointerv Surg. (2016) 8:418–22. 10.1136/neurintsurg-2014-011562 25676148

[23] Fox G Gallacher D Shevde S Loftus J Swayne G. Anatomic variation of the middle cerebral artery in the Sprague-Dawley rat. Stroke. (1993) 24:2087–92; discussion 2092–83. 10.1161/01.STR.24.12.2087 8248992

[24] Niiro M Simon RP Kadota K Asakura T. Proximal branching patterns of middle cerebral artery (MCA) in rats and their influence on the infarct size produced by MCA occlusion. J Neurosci Methods. (1996) 64:19–23. 10.1016/0165-0270(95)00058-5 8869480

[25] Qian B Rudy RF Cai T Du R. Cerebral artery diameter in inbred mice varies as a function of strain. Front Neuroanat. (2018) 12:10. 10.3389/fnana.2018.00010 29515376 PMC5826213

[26] Oliff HS Weber E Miyazaki B Marek P. Infarct volume varies with rat strain and vendor in focal cerebral ischemia induced by transcranial middle cerebral artery occlusion. Brain Res. (1995) 699:329–31. 10.1016/0006-8993(95)01045-W 8616638

[27] Bardutzky J Shen Q Henninger N Bouley J Duong Timothy Q Fisher M. Differences in ischemic lesion evolution in different rat strains using diffusion and perfusion imaging. Stroke. (2005) 36:2000–5. 10.1161/01.STR.0000177486.85508.4d 16040589 PMC2949947

[28] Walberer M Stolz E Müller C Friedrich C Rottger C Blaes F. Experimental stroke: ischaemic lesion volume and oedema formation differ among rat strains (a comparison between Wistar and Sprague-Dawley rats using MRI). Lab Anim. (2006) 40:1–8. 10.1258/002367706775404426 16460584

[29] Shah D Deleye S Verhoye M Staelens S Van Der Linden A. Resting-state functional MRI and [18F]-FDG PET demonstrate differences in neuronal activity between commonly used mouse strains. Neuroimage. (2016) 125:571–7. 10.1016/j.neuroimage.2015.10.073 26520769

[30] Kunze A Zierath D Drogomiretskiy O Becker K. Strain differences in fatigue and depression after experimental stroke. Transl Stroke Res. (2014) 5:604–11. 10.1007/s12975-014-0350-1 24916273

[31] Kunze A Zierath D Drogomiretskiy O Becker K. Variation in behavioral deficits and patterns of recovery after stroke among different rat strains. Transl Stroke Res. (2014) 5:569–76. 10.1007/s12975-014-0337-y 24711015

[32] Platt SR Holmes SP Howerth EW Duberstein KJJ Dove CR Kinder HA. Development and characterization of a Yucatan miniature biomedical pig permanent middle cerebral artery occlusion stroke model. Exp Transl Stroke Med. (2014) 6:5. 10.1186/2040-7378-6-5 24655785 PMC3977938

[33] Kaiser EE West FD. Large animal ischemic stroke models: replicating human stroke pathophysiology. Neural Regen Res. (2020) 15:1377–87. 10.4103/1673-5374.274324 31997796 PMC7059570

[34] Smith SM Jenkinson M Woolrich MW Beckmann CF Behrens TE Johansen-Berg H. Advances in functional and structural MR image analysis and implementation as FSL. Neuroimage. (2004) 23 (Suppl. 1):S208–19. 10.1016/j.neuroimage.2004.07.051 15501092

[35] Woolrich MW Jbabdi S Patenaude B Chappell M Makni S Behrens T. Bayesian analysis of neuroimaging data in FSL. Neuroimage. (2009) 45:S173–86. 10.1016/j.neuroimage.2008.10.055 19059349

[36] Jenkinson M Beckmann CF Behrens TE Woolrich MW Smith SM. Fsl. Neuroimage. (2012) 62:782–90. 10.1016/j.neuroimage.2011.09.015 21979382

[37] Rosset A Spadola L Ratib O. Osirix: an open-source software for navigating in multidimensional DICOM images. J Digital Imaging. (2004) 17:205–16. 10.1007/s10278-004-1014-6 15534753 PMC3046608

[38] Zhang YY Brady M Smith SA. Segmentation of brain MR images through a hidden markov random field model and the expectation maximization algorithm. IEEE Transact Med Imaging. (2001) 20:45–57. 10.1109/42.906424 11293691

[39] Kazemi K Noorizadeh N. Quantitative comparison of SPM, FSL, and Brainsuite for brain MR image segmentation. J Biomed Phys Eng. (2014) 4:13–26. 25505764 PMC4258855

[40] Schindelin J Arganda-Carreras I Frise E Kaynig V Longair M Pietzsch T. Fiji: an open-source platform for biological-image analysis. Nat Methods. (2012) 9:676–82. 10.1038/nmeth.2019 22743772 PMC3855844

[41] Gerriets T Stolz E Walberer M Müller C Kluge A Bachmann A. Noninvasive quantification of brain edema and the space-occupying effect in rat stroke models using magnetic resonance imaging. Stroke. (2004) 35:566–71. 10.1161/01.STR.0000113692.38574.57 14739415

[42] Loubinoux I Volk A Borredon J Guirimand S Tiffon B Seylaz J. Spreading of vasogenic edema and cytotoxic edema assessed by quantitative diffusion and T2 magnetic resonance imaging. Stroke. (1997) 28:419–26; discussion 426–7. 10.1161/01.STR.28.2.419 9040700

[43] Walmsley GL Herrtage ME Dennis R Platt SR Jeffery ND. The relationship between clinical signs and brain herniation associated with rostrotentorial mass lesions in the dog. Vet J. (2006) 172:258–64. 10.1016/j.tvjl.2005.05.012 16014332

[44] Lewis MJ Olby NJ Early PJ Mariani CL Munana KR Seiler GS. Clinical and diagnostic imaging features of brain herniation in dogs and cats. J Vet Intern Med. (2016) 30:1672–80. 10.1111/jvim.14526 27616749 PMC5032863

[45] Gerriets T Walberer M Ritschel N Tschernatsch M Mueller C Bachmann G. Edema formation in the hyperacute phase of ischemic stroke. J Neurosurg. (2009) 111:1036. 10.3171/2009.3.JNS081040 19408985

[46] Borich MR Wadden KP Boyd LA. Establishing the reproducibility of two approaches to quantify white matter tract integrity in stroke. NeuroImage. (2012) 59:2393–400. 10.1016/j.neuroimage.2011.09.009 21945470 PMC3249015

[47] Mahmoud BE Mohammad ME Serour DK What can DTI add in acute ischemic stroke patients? Egypt J Radiol Nucl Med. (2019) 50:67 10.1186/s43055-019-0058-z

[48] Swindle M Swine in the Laboratory: Surgery, Anesthesia, Imaging, and Experimental Techniques. Boca Raton: CRC Press (2007).

[49] Wang JC Hathorn I Habib AR Chang E Javer AR. Evaluation of domestic and Yucatan swine nasal sinus anatomy as models for future sinonasal research of medications delivered by standard instruments used in functional endoscopic sinus surgery. Int Forum Allergy Rhinol. (2013) 3:150–6. 10.1002/alr.21081 23038683

[50] Owen J Dobney K Evin A Cucchi T Larson G Strand Vidarsdottir U The zooarchaeological application of quantifying cranial shape differences in wild boar and domestic pigs (Sus scrofa) using 3D geometric morphometrics. J Archaeol Sci. (2014) 43:159–67. 10.1016/j.jas.2013.12.010

[51] Fawcett A Barrs V Awad M Child G Brunel L Mooney E. Consequences and management of canine brachycephaly in veterinary practice: perspectives from australian veterinarians and veterinary specialists. Animals. (2018) 9:3. 10.3390/ani9010003 30577619 PMC6356869

[52] Li S Francisco GE Zhou P. Post-stroke hemiplegic gait: new perspective and insights. Front Physiol. (2018) 9:1021. 10.3389/fphys.2018.01021 30127749 PMC6088193

[53] Kringe L Sena ES Motschall E Bahor Z Wang Q Herrmann AM. Quality and validity of large animal experiments in stroke: a systematic review. J Cereb Blood Flow Metab. (2020) 39:375–94. 10.1177/0271678X20931062 32576074 PMC7585919

