# Supplementary material for: Magnetic Resonance Imaging and Gait Analysis Indicate Similar Outcomes Between Yucatan and Landrace Porcine Ischemic Stroke Models
Source: Front Neurol. 2021 Jan 21;11:594954. doi: 10.3389/fneur.2020.594954 (PMC7859633; doi:10.3389/fneur.2020.594954)
Supplement: Supplementary file 3 [file Data_Sheet_1.PDF]

Supplementary Material

Manual ROI FA analysis

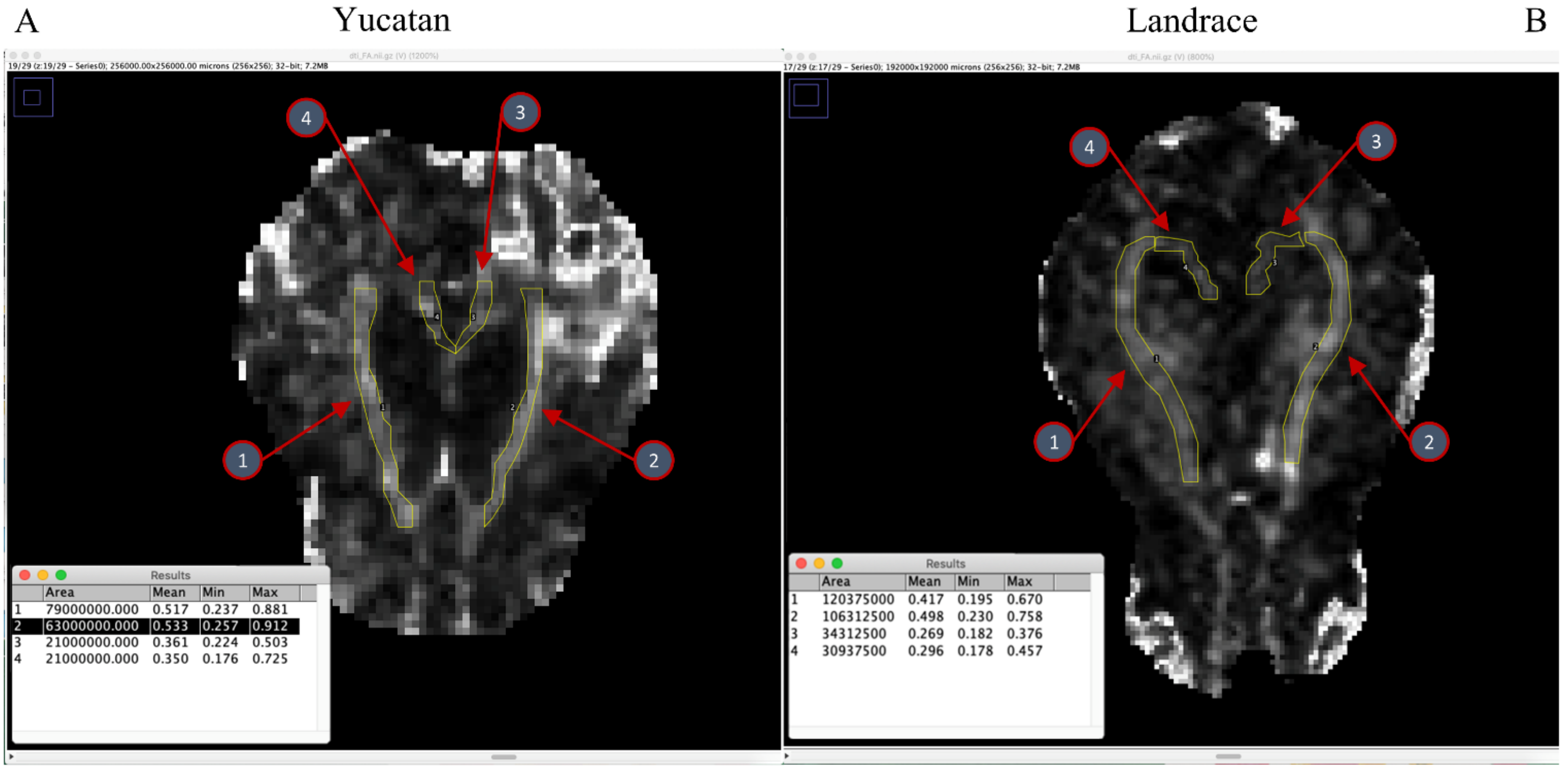

Supplementary Figure 1. Representative images of manual ROI analysis on pre-stroke MRI used to determine FA values for YC (**A**) and LR (**B**) animals. Red circled numbers correspond with the raw data values found in the bottom left corner of each panel. 1-RIC, 2-LIC, 3-LCC, and 4-RCC.

Animal Analysis Assignment and Use

Supplementary Table 1. The following table shows animal identification and study use.

Animal ID	Breed	Stroke	Pre-MRI	24hr-MRI	Pre-gait	2day-gait
**A1382**	YC	X	X			
**A1394**	YC	X	X	X	X	X
**A1401**	YC	X	X			
**A1422**	YC	X	X	X	X	X
**A1532**	YC	X	X	X	X	X
**A1589**	YC	X	X	X		
**A1604**	YC	X		X	X	X
**A1645**	YC	X	X			
**A1647**	YC	X	X	X	X	X
**A1653**	YC	X		X	X	X
**346**	LR		X			
**697**	LR		X			
**1R478**	LR	X		X	X	X
**2R40**	LR	X		X	X	X
**3R489**	LR	X		X	X	X
**4R495**	LR	X		X	X	X
**7R131**	LR	X		X	X	X
**9R137**	LR	X		X	X	X
**1W174**	LR	X	X	X		
**2W278**	LR		X			
**3W337**	LR	X		X		
**4W760**	LR	X		X		
**5W292**	LR	X		X		

Gait Collection Supplementary movies - Please see uploaded MP4 files.

Movie 1. This video depicts what researchers will observe using the GAITfour software during collection as the pig is travelling down the mat. Hoof falls are shown in the center of the screen (white box) as they transverse the mat with individual sensor data shown for each hoof in blue/green boxes. Using this pressure map of the hooves and collected video, researchers can discern if a run falls within the inclusion criteria.

Movie 2. This video depicts what is classified as a “good” run. The animal is shown to be travelling at a consistent trot. The video has been slowed to highlight the distinct gait of the pig.
